# Supplementary material for: A Cross-Sectional Survey of Salty Snack Consumption among Serbian Urban-Living Students and Their Contribution to Salt Intake
Source: Nutrients. 2020 Oct 27;12(11):3290. doi: 10.3390/nu12113290 (PMC7692209; doi:10.3390/nu12113290)
Supplement: Supplementary file 1 [file nutrients-12-03290-s001.pdf]

Questionnaire used in the study

Four letter code :

1. General questions:

Gender:

Year of bearth:

University student High-school student (circle the right answer)

Body mass (kg):

Body height (cm):

Examples of salt snack products (used in separate eating occasions): chips, flips, popcorn, fried corn, salted sticks, pretzels, crackers, fish-shaped products, roasted and baked nuts, seeds, and salty peanuts.

2. How often do you consume salty snack products?

never 1-3 x month 1 nedeljno 2-4 x week 1 daily several times per day

3. Where do you most often consume salty snacks?

At home At school /Faculty Outdoors

4. What is your preferable salty snack products? (write the snack type)

5. When do you usually consume salty snacks?

A. Before the main meal

During the main meal

Immediately after the main meal

Instead of the main meal

Between the main meals

B. What is your preferable part of day for salty snack consumption?

In the morning	Never	Sometimes	Often
In the afternoon	Never	Sometimes	Often
In the evening	Never	Sometimes	Often

6. What is the main characteristic that attracts you to these products?

**T**aste	Satiety potential
**P**ackage	No other food available at the moment
Crunchiness	Commercials
Easy availability	Other reasons (write them please)

7. Do you pay attention to the nutritive value of these products?

Yes, I read the information on the labels

No, I do not read information on the labels

8. Do you consume salty snack in front of TV or computer?

Often Sometimes Never

9. Do you often drink beverages with salty snacks?

No Yes, soft drinks Yes, drinks with alcohol

10. Do you change your habits in salty snacks consumption during periods of intensive learning?

No Yes, I use them more Yes, I use them less

11. How many daily meals do you usually have?

Main meals (write the number)

Refreshments (write the number)

12. What is your overall opinion about the quality of your diet?

Very bad Bad Good Very good

Supplementary Table S1.- Descriptive characteristics of study participants.

Parameter	University students N=806	High school students N=507	p
**Age, years**	23.0±2.4	17.3±1.3	< .001
**Male**	N=202 (25%)	N=160 (31.6%)	χ^2=6.8
**Female**	N=605 (75%)	N=346 (68.4%)	< .01
**BMI, kg/m²**	21.2±2.7	20.8±2.5	< .01
**<25 kg/m²**	91.4%	94.7%	
**25-30**	7.9%	4.7%	
**>30**	0.7%	0.6%	

p = ANOVA with post-hoc Tukey test, or Chi-square test.

Supplementary Table S2. - The rotated pattern matrix of the questionnaire scale. The four factors and their items.

**1. Pattern of salty snack consumption**	
**Dominant place of salty snack consumption**	0.950
Frequency of salty snack consumption	0.911
Time of salty snack consumption (regarding main meal)	0.813
SP type	0.787
2. **Information about nutritive value**	
Information on food labels	0.747
3. **General dietary habits**	
Number of main meals	0.821
Number of refreshments	0.720
Eating with nonalcoholic or alcoholic beverages	0.595
Eating in front of TV	0.384
**4. Preferences**	
Faculty/high school	0.796
Motivational reasons for salty snack consumption	-0.561
Day period related consumption	0.342

Supplementary Table S3. Logistic regression analysis of the variables (questionnaire's items) that could predict a high level of salty snack product intake among the population of urban living students.

Population	All				High-school students				University students			
**Parameter/Data**	**β (SE)**	**Wald**	**OR 95th CI**	***p***	**β (SE)**	**Wald**	**OR 95th CI**	***p***	**β (SE)**	**Wald**	**OR 95th CI**	***p***
**Gender m/f**	0.016 (0.221)	0.01	1.016 (0.659-1.565)	0.943	0.268 (0.319)	0.70	1.307 (0.700-2.442)	0.401	-0.189 (0.306)	0.382	0.828 (0.454-1.509)	0.537
**Age (years)**	**-0.122 (0.031)**	**16.0**	**0.885 (0.833-0.939)**	**<0.001**	-0.328 (0.111)	8.67	0.721 (0.579-0.896)	0.003	-0.053 (0.060)	0.777	0.948 (0.843-1.067)	0.378
**BMI**	-0.024 (0.037)	0.424	0.976 (0.907-1.050)	0.515	-0.042 (0.057)	0.54	0.959 (0.857-1.073)	0.464	0.010 (0.049)	0.044	1.010 (0.918-1.112)	0.834
**Type of snack products**	**-0.320 (0.051)**	**32.5**	**0.780 (0.700-0.853)**	**< 0.001**	**0.260 (0.064)**	**22.4**	**0.816 (0.796-0.856)**	**<0.001**	**-0.374 (0.063)**	**18.5**	**0.922 (0.867-0.980)**	**<0.001**
**Awareness of SP intake**	0.507 (0.220)	5.3	1.660 (1.081-2.550)	0.021	0.192 (0.313)	0.38	1.212 (0.655-2.239)	0.540	0.688 (0.314)	4.81	1.991 (1.076-3.682)	0.028
**Morning consumption frequency**	**1.091 (0.174)**	**39.4**	**2.98 (2.121-4.182)**	**<0.001**	**1.690 (0.286)**	**35.18**	**5.464 (3.119-9.589)**	**<0.001**	0.729 (0.237)	9.47	2.074 (1.303-3.300)	0.002
**Midday consumption frequency**	**1.550 (0.191)**	**56.7**	**4.71 (3.240-6.845)**	**<0.001**	**1.593 (0.285)**	**31.23**	**4.916 (2.812-8.594)**	**<0.001**	**1.498 (0.263)**	**32.46**	**4.473 (2.672-7.490)**	**<0.001**
**Evening consumption frequency**	**1.40 (0.177)**	**63.0**	**4.074 (2.886-5.753)**	**<0.001**	**1.056 (0.231)**	**20.92**	**2.876 (1.829-4.522)**	**<0.001**	**1.981 (0.302)**	**43.03**	**7.252 (4.012-13.110)**	**<0.001**
**Motivational reasons**	0.001 (0.000)	1.74	1.000 (1.000-1.001)	0.187	0.001 (0.001)	1.35	1.001 (1.000-1.002)	0.245	0.000 (0.001)	0.01	1.000 (0.999-1.002)	0.932
**Number of main meals**	0.264 (0.168)	2.45	1.303 (0.936-1.812)	0.117	0.272 (0.247)	1.21	1.313 (0.809-2.131)	0.271	0.173 (0.253)	0.54	1.189 (0.749-1.885)	0.436
**Number of refreshments**	**0.560 (0.097)**	**33.4**	**1.751 (1.451-2.122)**	**<0.001**	**0.473 (0.117)**	**16.44**	**1.605 (1.277-2.018)**	**<0.001**	0.577 (0.185)	9.67	1.780 (1.238-2.561)	0.002
**Self-perception of overall diet quality**	**-0.636 (0.161)**	**15.7**	**0.529 (0.386-0.725)**	**<0.001**	**-1.011 (0.239)**	**17.96**	**0.364 (0.288-0.581)**	**<0.001**	-0.313 (0.234)	1.78	0.732 (0.462-1.158)	0.182

*SP = salty snack products; OR-odds ratio coefficient (95th CI- confidence interval).
